# A Predominant Variable-Number Tandem-Repeat Cluster of Mycobacterium tuberculosis Isolates among Asylum Seekers in the Netherlands and Denmark, Deciphered by Whole-Genome Sequencing

**DOI:** 10.1128/JCM.01100-17

**Published:** 2018-01-24

**Authors:** Rana Jajou, Albert de Neeling, Erik Michael Rasmussen, Anders Norman, Arnout Mulder, Rianne van Hunen, Gerard de Vries, Walid Haddad, Richard Anthony, Troels Lillebaek, Wim van der Hoek, Dick van Soolingen

**Affiliations:** a National Institute for Public Health and the Environment, Bilthoven, The Netherlands; b International Reference Laboratory of Mycobacteriology, Statens Serum Institut, Copenhagen, Denmark; c KNCV Tuberculosis Foundation, The Hague, The Netherlands; d Municipal Health Service, GGD Hart voor Brabant, ‘s-Hertogenbosch, The Netherlands; e Radboud University Medical Center, Department of Medical Microbiology, Nijmegen, The Netherlands; Johns Hopkins University School of Medicine

**Keywords:** Mycobacterium tuberculosis, asylum seekers, The Netherlands, Denmark, VNTR, WGS

## Abstract

In many countries, Mycobacterium tuberculosis isolates are routinely subjected to variable-number tandem-repeat (VNTR) typing to investigate M. tuberculosis transmission. Unexpectedly, cross-border clusters were identified among African refugees in the Netherlands and Denmark, although transmission in those countries was unlikely. Whole-genome sequencing (WGS) was applied to analyze transmission in depth and to assess the precision of VNTR typing. WGS was applied to 40 M. tuberculosis isolates from refugees in the Netherlands and Denmark (most of whom were from the Horn of Africa) that shared the exact same VNTR profile. Cluster investigations were undertaken to identify in-country epidemiological links. Combining WGS results for the isolates (all members of the central Asian strain [CAS]/Delhi genotype), from both European countries, an average genetic distance of 80 single-nucleotide polymorphisms (SNPs) (maximum, 153 SNPs) was observed. The few pairs of isolates with confirmed epidemiological links, except for one pair, had a maximum distance of 12 SNPs. WGS divided this refugee cluster into several subclusters of patients from the same country of origin. Although the M. tuberculosis cases, mainly originating from African countries, shared the exact same VNTR profile, most were clearly distinguished by WGS. The average genetic distance in this specific VNTR cluster was 2 times greater than that in other VNTR clusters. Thus, identical VNTR profiles did not represent recent direct M. tuberculosis transmission for this group of patients. It appears that either these strains from Africa are extremely conserved genetically or there is ongoing transmission of this genotype among refugees on their long migration routes from Africa to Europe.

## INTRODUCTION

Tuberculosis (TB) is caused by Mycobacterium tuberculosis and is the leading worldwide cause of death from a single pathogen (1). The Netherlands is a country with low incidence; between 1994 and 2014, the incidence of TB decreased from 11.7 to 4.9 cases per 100,000 inhabitants. However, due to the increasing number of immigrants and asylum seekers from countries with high incidences of TB, the TB incidence increased to 5.1 cases per 100,000 inhabitants in 2015. Of all 867 patients diagnosed in 2015, 625 (72.1%) were born outside the Netherlands, with the majority of TB patients originating from Eritrea, Somalia, and Morocco (2). The increase in immigrants from countries with higher TB incidences is also an important issue in other European countries with low incidences. In the Nordic countries, for instance, a considerable influx of immigrants, which has contributed significantly to the TB incidence in those countries, has been reported (3–6). In Denmark, for example, the overall TB incidence in 2015 was 6.3 cases per 100,000 inhabitants, with 75% of cases being of non-Danish origin; 19% of the cases were from the Horn of Africa (7).

DNA fingerprinting of all M. tuberculosis isolates, to monitor TB transmission, has been structurally applied in the Netherlands since 1993 and in Denmark since 1992 and is performed by the (inter)national tuberculosis reference laboratories in the two countries. IS*6110* restriction fragment length polymorphism (RFLP) typing was the first standard for typing and was applied from 1993 to 2008 in the Netherlands and from 1992 to 2006 in Denmark. In 2009, 24-locus variable-number tandem-repeat (VNTR) typing was introduced as the new standard in the Netherlands, in line with international developments (8), with retrospective retyping of all isolates from 2004 to 2008. The concordance between the two methods for the isolates typed by both RFLP and VNTR typing was described earlier (9). In Denmark, the switch to VNTR typing took place in 2007, with retrospective retyping of the isolates from 2003 to 2006.

The municipal health services (MHSs) in the Netherlands receive information on clustering of DNA fingerprints for M. tuberculosis isolates on a weekly basis, to guide investigations of epidemiological relationships and transmission patterns. In Denmark, investigations based on epidemiological links are not as systematically registered, and only limited linkage information is available.

Already in 1993, the RFLP pattern associated with cluster 15 was detected in the northern part of the Netherlands; it involved a 20-year-old patient from Somalia who was assumed to suffer from endogenous reactivation of a latent infection contracted before arrival in the Netherlands. Over the years, this cluster expanded throughout the Netherlands, with ≥2 new cases per year. The change in typing methodology transformed RFLP cluster 15 into VNTR cluster 1064-32, which has also been identified in Denmark. In the Netherlands, 45 isolates from the period of 2004 to 2008 were assigned to cluster 1064-32. Full concordance between the two DNA fingerprinting methods was found for 14/45 isolates (31%), meaning that these isolates had exactly the most prevalent profile of RFLP cluster 15 and VNTR cluster 1064-32. Another 14/45 isolates (31%) presented with a slightly different VNTR profile but the same RFLP pattern (pattern 15). The remaining 17/45 isolates (38%) demonstrated VNTR profile 1064-32 but slightly different RFLP patterns.

Because an unexpectedly high degree of VNTR clustering among refugees and immigrants occurred in both the Netherlands and Denmark, whole-genome sequencing (WGS) was applied to isolates belonging to asylum seekers VNTR cluster 1064-32 from both countries. The mainly noninfectious character of the M. tuberculosis in asylum seekers from several countries and the low level of confirmed epidemiological links make transmission in the Netherlands unlikely and brings into question the specificity of clustering on the basis of RFLP/VNTR profiles in this specific cluster. Based on two previous studies (5, 10), a similar conclusion was reached for the majority of cases in the refugee cluster in Denmark. This suggests that (i) transmission occurred on the migration route or (ii) there is a predominant genotype of M. tuberculosis with a high degree of genetic conservation circulating in the countries of origin. In this study, the high resolution of WGS and detailed information on patient characteristics were combined to address the confusion in the molecular epidemiology of TB in refugees. If part of the clustering on basis of VNTR typing in this group of patients is resolved through WGS analysis, then recent transmission rates may be overestimated.

## MATERIALS AND METHODS

### Patient selection.

Using the most common VNTR profile of cluster RFLP 15/VNTR 1064-32 (24-locus VNTR pattern in the order of VNTR154, VNTR424, VNTR577, VNTR 580, VNTR802, VNTR960, VNTR1644, VNTR1955, VNTR2059, VNTR2163b, VNTR2165, VNTR2347, VNTR2401, VNTR2461, VNTR2531, VNTR2687, VNTR2996, VNTR3007, VNTR3171, VNTR3192, VNTR3690, VNTR4052, VNTR4156, VNTR4348; 242247432244225113342543), 12 of the 205 patient isolates from the Netherlands were selected to obtain variations in the country of birth, the duration of living in the Netherlands (<6 months versus ≥6 months), the diagnosis of multidrug-resistant (MDR)-TB, and epidemiological links with another patient in the cluster. Patient characteristics were obtained from the Netherlands Tuberculosis Register (NTR), and information on epidemiological links was obtained from the respective MHS. From Denmark, isolates from 31 patients shared the VNTR 1064-32 profile and were all subjected to WGS. The samples from these patients were collected in the period from 2001 through 2016.

### DNA purification.

The M. tuberculosis isolates from the Netherlands were regrown in mycobacterial growth indicator tube (MGIT) tubes from −70°C freezer stocks, except for the isolates from 2016, which were directly available. DNA was isolated and purified with the QIAamp DNA minikit (Qiagen GmbH, Hilden, Germany) method, after the samples were incubated with lysis buffer and proteinase K to release the DNA (11). M. tuberculosis isolates from Denmark were regrown in MGIT tubes from −80°C freezer stocks. One milliliter was taken from a positive MGIT culture and centrifuged. DNA was isolated according to a previously reported method (12).

### Read mapping and variant calling.

The isolates from the Netherlands were sequenced with an Illumina HiSEQ2500 sequencer and the isolates from Denmark were sequenced with an Illumina NextSeq system, following the Nextera XT protocol, according to the manufacturer's instructions. Breseq software (version 0.28.1) was used to map the reads to the H37Rv reference genome (version 3.0; GenBank accession no. AL123456 or Refseq accession no. NC_000962) and to call single-nucleotide polymorphisms (SNPs) supported by ≥80% of the mapped reads in regions with coverage of ≥5 reads. Fastq files for the isolates from the Netherlands were uploaded to PhyResSe (https://bioinf.fz-borstel.de/mchips/phyresse) to assign a lineage to each isolate. For the isolates from Denmark, the SNP barcode system proposed by Coll et al. (13) was used to assign the isolates to a lineage. This method detected the G/A SNP at position 1,084,911 in all isolates, which identified them as lineage 3.1.1, i.e., central Asian strain (CAS)/Delhi. Called variants were compared to a set of global reference strains belonging to the same lineage (ENA accession no. ERP001731).

### Data analysis.

WGS data analysis was performed in R Statistics (version 3.2). SNPs annotated as PE/PPE, PGRS, esx, repeat, polyketide, pks, or transposase were excluded during WGS data analysis. Squared Euclidean distance matrices were generated to analyze the genetic distance in SNPs between isolates. A minimum spanning tree was built using Ridom SeqSphere+ software (version 4.0; Ridom GmbH, Münster, Germany) to visualize how isolates from the Netherlands and Denmark were clustered according to WGS, using a cutoff maximum of 12 SNPs (14). All isolates from the Netherlands cultured between 1 January 2016 and 31 December 2016 were subjected to both VNTR typing and WGS. Therefore, it was possible to compare the average genetic distance between isolates in cluster 1064-32 and isolates belonging to other VNTR clusters. The phenotypic resistance profile of the MDR-TB patients was verified on the basis of detected mutations in resistance genes using the PhyResSe pipeline. Phenotypic drug susceptibility testing (DST) was performed with a MGIT 960 system.

## RESULTS

### Description of the clusters.

**FIG 1:**
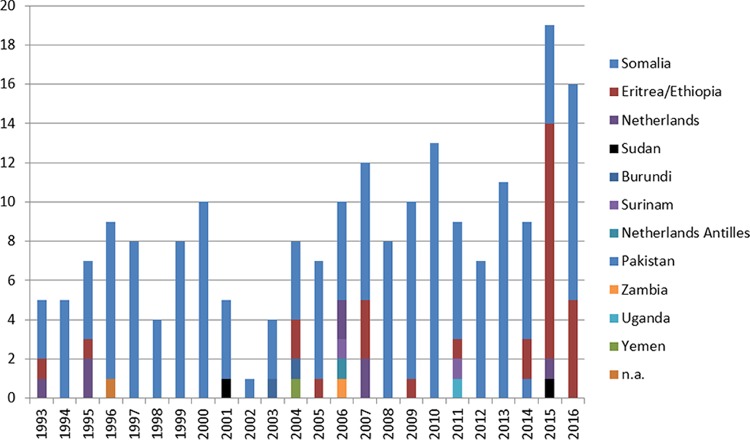
Cluster RFLP 15/VNTR 1064-32 in the Netherlands, according to origin. n.a., not applicable.

Cluster 1064-32 in the Netherlands included 205 patients in 2016, of whom 156 (76.1%) were born in Somalia, 29 (14.1%) in Eritrea/Ethiopia, 8 (3.9%) in the Netherlands, 2 (1%) in Sudan, 2 (1%) in Burundi, 2 (1%) in Surinam, 1 (0.5%) in the Netherlands Antilles, 1 (0.5%) in Pakistan, 1 (0.5%) in Zambia, 1 (0.5%) in Uganda, and 1 (0.5%) in Yemen; the origin was not registered for 1 patient (0.5%) (Fig. 1). Among those 205 patients, 102 were asylum seekers, of whom 63.7% (65/102 patients) had TB diagnosed within 2.5 years after arriving in the Netherlands. Epidemiological links were confirmed for only 3.9% of the cases (8/205 cases); 2 cases were identified by RFLP typing and 6 by VNTR typing. Fifty-four percent of the patients (111/205 patients) were male. Seventy patients (34.1%) had pulmonary tuberculosis (PTB) only, 33 (16.1%) had a combination of PTB and extrapulmonary tuberculosis (EPTB), and 102 (49.8%) had EPTB only. Data on DST for first-line antibiotics showed that 66.8% of the isolates (137/205 iolates) were fully sensitive, 15 isolates (7.3%) were monoresistant to isoniazid (INH), 4 isolates (2%) were resistant to INH and rifampin (RIF) (i.e., MDR-TB), 2 isolates (1%) were monoresistant to RIF, and DST results were missing from the NTR for the remaining 47 isolates (22.9%).

Twelve of the 205 isolates from patients in the Netherlands were included in this study, including 8 patients from Somalia, 3 patients from Eritrea (2 of whom were epidemiologically linked), and 1 patient from the Netherlands. Four Somalian patients from 2016 were from one family, in which the source case (patient NL16-10) was suspected to have infected three family members (patients NL16-09, NL16-11, and NL16-12), based on MHS data (Table 1). Of the 12 included isolates, 2 were MDR, 2 were streptomycin (STR) resistant, 4 were susceptible, and DST data were not available in the NTR for the remaining 4 isolates.

**TABLE 1:** Characteristics of the 40 included M. tuberculosis patients from the Netherlands and Denmark

Patient	Gender	Age when clinical sample obtained (yr)	Year of diagnosis	Country of birth	Epidemiological link with:	Comments
NL05-01	M	36	2005	Somalia		≥6 mo in the Netherlands
NL06-02	M	26	2006	Somalia		<6 mo in the Netherlands; RFLP 276
NL11-03	M	22	2011	Eritrea		MDR
NL13-04	F	10	2013	Somalia		MDR
NL14-05	M	25	2014	Eritrea	NL14-06	
NL14-06	M	24	2014	Eritrea	NL14-05	
NL15-07	M	23	2015	Netherlands		
NL15-08	F	28	2015	Somalia		<6 mo in the Netherlands
NL16-09	M	73	2016	Somalia	NL16-10	
NL16-10	F	20	2016	Somalia	NL16-09, NL16-11, NL16-12	
NL16-11	M	17	2016	Somalia	NL16-10	
NL16-12	F	46	2016	Somalia	NL16-10	
DK15-01	M	32	2015	Unknown		
DK16-03	M	28	2016	Eritrea		
DK15-04	M	27	2015	Eritrea		
DK01-05	F	21	2001	Somalia		MDR
DK16-06	M	22	2016	Eritrea		
DK14-07	M	19	2014	Somalia		Rifampin monoresistant
DK14-08	F	20	2014	Somalia		
DK03-09	M	32	2003	Somalia		
DK05-11	M	14	2005	Somalia		
DK16-12	M	29	2016	Eritrea		
DK06-14	M	6	2006	Somalia	DK06-29	
DK06-15	F	43	2006	Somalia		
DK07-16	F	27	2007	Uganda		
DK07-17	F	17	2007	Somalia		
DK05-18	F	30	2005	Somalia		
DK05-19	M	21	2005	Eritrea		
DK04-20	M	44	2004	Somalia		
DK09-21	F	19	2009	Somalia		
DK16-22	F	29	2016	Eritrea		
DK15-23	M	27	2015	Somalia		
DK16-24	F	20	2016	Eritrea		
DK15-25	M	52	2015	Denmark		
DK14-26	M	20	2014	Denmark		Somalia-born parents
DK16-27	F	33	2016	Eritrea		
DK06-29	M	42	2006	Somalia	DK06-14	
DK06-30	M	20	2006	Somalia		
DK13-31	M	28	2013	Somalia		
DK15-32	M	30	2015	Eritrea		

**FIG 2:**
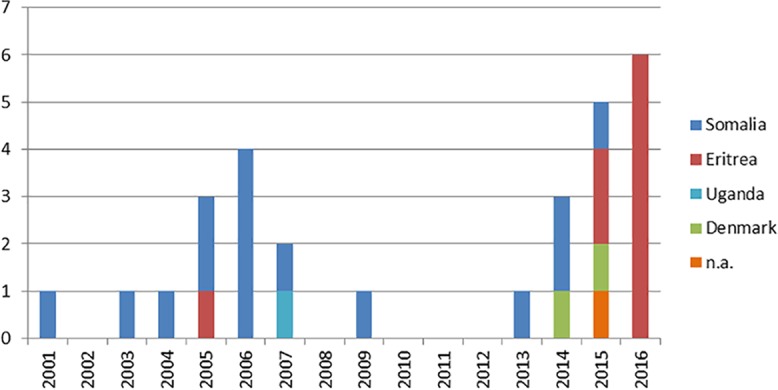
Cluster VNTR 1064-32 in Denmark, according to origin. n.a., not applicable.

A cluster in Denmark with the same VNTR pattern included 31 cases, of which 3 cases were excluded due to low levels of coverage of the sequenced isolates, leaving 28 strains available for inclusion in this study (Table 1). Of those 28 cases, 15 (53.6%) originated from Somalia, 9 (32.1%) from Eritrea, 2 (7.1%) from Denmark, and 1 (3.6%) from Uganda; 1 case (3.6%) was of unknown country of origin (Fig. 2). One of the 2 Denmark-born patients had Somalian parents. Sixty-four percent of the patients (18/28 patients) were male. Ten patients (35.7%) had PTB only, 2 patients (7.1%) had a combination of PTB and EPTB, and 16 patients (57.1%) had EPTB only. Isolates from 26 patients (92.9%) were fully sensitive to all first-line antibiotics, and the remaining 2 isolates were RIF resistant, of which one was a MDR-TB isolate with additional resistance to ethambutol (EMB), pyrazinamide (PZA), roxithromycin (ROX), cycloserine (CYA), and capreomycin (CPM).

### WGS clustering of asylum seekers in the Netherlands and Denmark.

The genetic distances between the isolates from the Netherlands and Denmark, all belonging to the CAS/Delhi genotype, ranged from 1 to 153 SNPs; the degree of genetic conservation was comparable among the isolates from the Netherlands versus the isolates from Denmark. The average genetic distance among all 40 isolates in cluster 1064-32 was 80 SNPs, compared to 43 SNPs for isolates in other VNTR clusters in the Netherlands.

**FIG 3:**
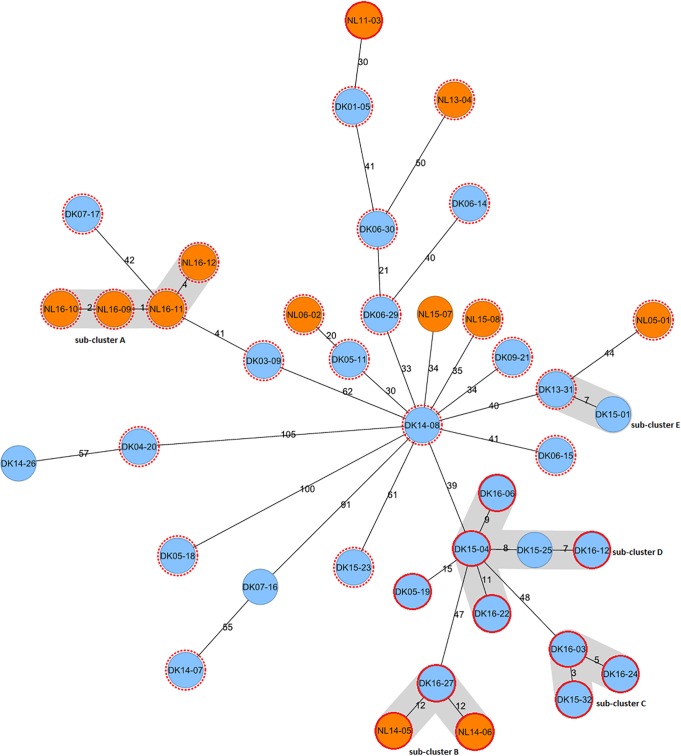
Minimum spanning tree of 40 M. tuberculosis isolates from asylum seekers in the Netherlands (orange) and Denmark (blue), belonging to the same VNTR cluster. No unknown epidemiological links were allowed. The numbers on the branches indicate the genetic distance (in SNPs) to the nearest isolate. SNP differences between distant strains cannot be reconstituted by summing the number of SNPs on the branches. Red dotted lines represent cases of Somalian origin, and red solid lines represent cases of Eritrean origin; the first two digits in the case numbers indicate the year of TB diagnosis. Subclustering on the basis of WGS (≤12-SNP distance between isolates) is shown in gray.

WGS divided the isolates from cluster 1064-32 into several subclusters. All subclusters consisted of patients from the same country of origin, except for subcluster D, in which a native Danish case (patient DK15-25) clustered with two cases from Eritrea (patients DK15-04 and DK16-12) (Fig. 3). The 6 isolates from patients in the Netherlands with confirmed epidemiological links had a genetic distance of ≤12 SNPs and formed 2 of 5 WGS subclusters (subclusters A and B) (Fig. 3). The only identified epidemiological link for the cases from Denmark was between patient DK06-29 (father) and patient DK06-14 (son), with a genetic distance of 40 SNPs (Fig. 3). The genetic distance between isolates from patients without a confirmed epidemiological link ranged from 3 to 153 SNPs, meaning that some patients for whom epidemiological links could not be identified were clustered on the basis of WGS data (subclusters C, D, and E) (Fig. 3).

Five isolates from Denmark (patients DK14-07, DK07-16, DK05-18, DK04-20, and DK14-26) had much greater genetic distances than did almost all other isolates in the cluster. Patient DK14-26, a Denmark-born patient with Somalian parents, and patient DK04-20, who was Somalian, exhibited a distance of 57 SNPs from each other but varied by ≥105 SNPs from all other isolates. A similar situation was observed for patient DK14-07, originally from Somalia, and patient DK07-16, the only patient originally from Uganda, who were most related to each other but were ≥90 SNPs distant from all other isolates studied. The isolate from patient DK05-18 was ≥100 SNPs distant from all other included isolates. The genetic distance between the three MDR-TB isolates was a maximum of 59 SNPs; isolate NL11-03, of Eritrean origin, was more related to the Danish case of Somalian origin than to the MDR-TB case in the Netherlands of Somalian origin (Fig. 3).

### Resistance profiles of MDR-TB patients.

The two MDR-TB isolates from the Netherlands were both resistant to INH, RIF, and EMB. Isolate NL11-03 was also resistant to STR and PZA. Mutations in associated resistance genes were identified for all drugs except STR. The MDR-TB isolate from Denmark was resistant to RIF, INH, PZA, EMB, ROX, CYA, and CPM. This isolate had mutations in the resistance-associated *rpoB* (S450L), *katG* (S315T), *pncA* (A−11G), and *embB* (Q497R) genes (Table 2).

**TABLE 2:** Comparison of phenotypic DST and WGS results for the three MDR patients

Patient no.	RIF^*a*^			INH			PZA			EMB			STR	
	DST result	*rpoB*	Variant	DST result	*katG*	Variant	DST result	*pncA*	Variant	DST result	*embB*	Variant	DST result	*rpsL-gidB-rrs-tlyA*
NL11-03	R	S450L	C→T	R	S315T	G→C	R	W119R	T→C	R	Q497R	A→G	R	S
NL13-04	R	S450L	C→T	R	S315T	G→C	S	S	S	R	M306I	G→T	S	S
DK01-05	R	S450L	C→T	R	S315T	G→C	R	A−11G	A→G	R	Q497R	A→G	S	S

a RIF, rifampin; INH, isoniazid; PZA, pyrazinamide; EMB, ethambutol; STR, streptomycin; R, resistant; S, susceptible.

## DISCUSSION

This study showed that a large cluster of M. tuberculosis isolates, mostly from asylum seekers, shared the same VNTR profile but were not identical based on WGS analysis. WGS divided this VNTR cluster into several subclusters, with almost all subclusters including patients from the same country of origin. Moreover, the average genetic distance in this specific VNTR cluster was twice as large as those in other VNTR clusters. This supports the findings from epidemiological investigations, showing that transmission did not occur in the destination countries. Refugees originate from countries with wars and hunger and financial crises, resulting in sustained migration to European countries. The patients share migration routes to Europe, including long-term stays in large refugee camps, where TB transmission most likely occurred.

This hypothesis is supported by a recent multicountry cluster investigation of 28 migrant MDR-TB cases in Europe, in which it was suggested that cases were part of a chain of recent transmission that likely occurred in the country of origin or on the migration route (15). A similar study was performed in Switzerland, a country with low TB incidence like the Netherlands. The study reanalyzed VNTR clusters using WGS to assess TB transmission among native and foreign-born TB patients. The outcome of that study was comparable to our results, i.e., overestimation of TB transmission based on VNTR typing, compared with WGS, especially among immigrants from countries with high TB incidences (16).

Several other findings from this study support our hypotheses. In the Netherlands, 19% of the overall TB cases that were clustered by VNTR typing within a 2-year period were found by MHSs to be epidemiologically linked in 2014 (2). In the VNTR cluster described in this study, however, TB nurses interviewing the patients could hardly confirm any epidemiological links between cases. The low degree of confirmed epidemiological links identified by MHSs seems to be logical, as only nontransmissible EPTB was observed in the majority of cases. Also, the average genetic distance between the isolates in cluster 1064-32 was much greater than that in other clusters in the Netherlands, in which the degree of confirmation of epidemiological links is generally higher (17). Therefore, the benefits of WGS for epidemiological research among asylum seekers are even greater than for regular patients in these low-prevalence settings.

Screening programs in the Netherlands targeting asylum seekers/immigrants from countries with high TB incidences aim to detect TB disease in these populations. However, these screening programs focus only on active disease and not on migrants latently infected. In Denmark, asylum seekers are offered health interviews and, in cases with symptoms, additional testing can be performed. It is assumed that most asylum seekers who develop disease after arrival are experiencing an endogenous reactivation rather than a new infection due to recent transmission after arriving in the destination country (3, 18). Nevertheless, the possibility of transmission in the destination country remains. In WGS subcluster D for instance, a native Danish patient clustered with patients of Eritrean origin. No information was available about the potential source of infection for this native Danish patient. Although previous studies suggested that transmission of M. tuberculosis from immigrants to the native population is limited (5, 10, 19, 20), it is likely that such transmission did occur, since TB was diagnosed in 2015 or 2016 for all involved patients. Among the five native Dutch patients in cluster 1064-32, an epidemiological link with a patient from Zambia was confirmed for two, and one patient had worked in Africa. For the remaining two native Dutch patients, an epidemiological link with another patient in the cluster could not be found. Identifying epidemiological links among immigrants remains a challenge, due to cultural and language barriers or reluctance to reveal the real identities of family members and/or friends or actual travel histories.

For the six pairs of isolates from patients in the Netherlands with confirmed epidemiological links, the genetic distance was limited to ≤12 SNPs. In line with the criteria described by Walker et al. (14) to rule in possible epidemiological links, these cases were correctly clustered by VNTR typing. However, the two epidemiologically linked cases in Denmark were 40 SNPs distant from each other. The pace of genetic turnover may be faster in this strain, for unknown reasons. For the other nonlinked cases in both countries, genetic distances of only up to 153 SNPs were found. This is highly remarkable, as this suggests an extremely high degree of genetic conservation in this predominant genotype of M. tuberculosis circulating in a large geographic area. One way to investigate the reason for this high degree of conservation would be to sample M. tuberculosis in a representative way from TB patients in both Somalia and Eritrea (and possibly other countries), to study the actual population structures of M. tuberculosis and the degrees of genetic conservation within the countries of origin. Given the low discriminative power of the current methods for DNA typing in Western countries when analyzing cases from refugees, this seems a sensible investment that may be supported by larger health organizations, such as the World Health Organization (WHO) or the European Centre for Disease Prevention and Control (ECDC).

In conclusion, VNTR typing of M. tuberculosis isolates from African asylum seekers is misleading. When WGS was applied, several subclusters were identified, consisting mainly of patients from the same country of origin. Despite the fact that the average genetic distance within this cluster was twice as great as those for other VNTR clusters, the degree of genetic conservation within this diverse study population remains remarkable, suggesting that these cases are linked and transmission occurred during the migration or a highly conserved genotype is circulating in the refugees' countries of origin.
